# *Cryptococcus* spp. isolation from excreta of pigeons (*Columba livia*) in and around Monterrey, Mexico

**DOI:** 10.1186/2193-1801-2-632

**Published:** 2013-11-23

**Authors:** Yolanda Canónico-González, Juan Manuel Adame-Rodríguez, Roberto Mercado-Hernández, Elva Teresa Aréchiga-Carvajal

**Affiliations:** Laboratorio de Micología y Fitopatología, Departamento de Microbiología e Inmunología, Universidad Autónoma de Nuevo León, San Nicolás de los Garza, Mexico; Departamento de Ciencias Exactas Facultad de Ciencias Biológicas, Cd Universitaria, San Nicolás de los Garza, NL Mexico; Unidad de Manipulación Genética, Laboratorio de Micología y Fitopatología (LMYF), Facultad de Ciencias Biológicas, Segundo Piso, Unidad C Universidad Autónoma de Nuevo León, Ave. Pedro de Alba s/n cruz Miguel L Barragán, Cd. Universitaria, San Nicolás de los Garza, Nuevo León Mexico

**Keywords:** Cryptococcosis, *Neoformans*, *Albidus*, Pigeon (*Columba livia*), México

## Abstract

The presence of *Cryptococcus* spp. has been reported in Mexico’s capital city; however, to our knowledge there are no reports of its presence in the state of Nuevo León located in northeast Mexico. This is presumed to be because the hot and dry climate in this region does not favor cryptococcal proliferation. This study confirmed the presence of *C. neoformans* and *C. albidus* in 20% (10/50) of randomly selected fecal samples of pigeons (*Columba livia*) in the Monterrey metropolitan area. The presence of this yeast in the state of Nuevo León is proof of its adaptation to the typically hot climate of the area and is consistent with recent reviews of cryptococcosis cases in several local hospitals. The two species were identified and characterized through microbiological tests and molecular identification by DNA extraction and PCR amplification of highly conserved 18S ribosomal DNA using ITS1 and ITS2 as target regions. The PCR products were sequenced and compared with those reported in GenBank.

**Electronic supplementary material:**

The online version of this article (doi:10.1186/2193-1801-2-632) contains supplementary material, which is available to authorized users.

## Introduction

Thirty-eight yeast species within the *Cryptococcus* genus have been described, including *C. neoformans*, *C. gatti*, *C. albidus,* and *C. laurentii*, which have been extensively studied because of their clinical importance as etiologic agents of opportunistic cryptococosis (Rosario *et al.*^2008^; Castañón Olivares *et al.*^2000^). Cryptococcosis can result in serious meningitis and is acquired by inhalation of infective yeasts present in wood, fruits, rotting vegetables, soil, dairy products, and most frequently fecal material from urban pigeons (*Columba livia*) (Ellis and Pfeiffer, 1992; Khawcharoenporn *et al.*^2007^) that commonly accumulate in metropolitan areas where this species has successfully become established. This kind of mycosis is not considered endemic, mainly because there is only information about its presence in Mexico’s central region. The northeast region of the country has a dry and hot climate, where summer temperatures reach up to 40°C (between 35-40°C) even in the shade (Extreme temperatures and precipitation for Monterrey (OBS) 2000-2010 (in spanish) 2013). It has been suggested that these environmental conditions are not suitable for the growth of *Cryptococcus* spp. and that most strains cannot survive high temperatures because the majority of isolates reported are from tropical and subtropical countries where the average temperature is 22°C in the hottest month of the year (Lin and Heitman, 2006; Ishaq *et al.*^1968^; Ruiz *et al.*^1989^; Dromer *et al.*^1996^; Mancianti *et al.*^2002^; Rivas *et al.*^1999^; Quintero *et al.*^2005^; Curo *et al.*^2005^; Colom Valiente *et al.*^1997^; Castañol-Olivares and Lopez-Martinez, 1994; Casali *et al.*^2003^). Surprisingly, there have been 190 reports of cryptococcosis in six different state hospitals in Monterrey city from 1995 to 2011 (Casillas 2012). This could be evidence of its successful adaptation to the warm and dry climate present in this region. To investigate this we evaluated 50 random samples of pigeon droppings from three different Monterrey metropolitan locations.

## Materials and methods

Fifty fecal samples were collected in the summer between the months of August and September of 2010. We first considered the population density for each of the three municipal zones sampled (Guadalupe, Monterrey, and San Nicolás de los Garza) in the Monterrey metropolitan area in the state of Nuevo León. We obtained one sample for approximately every 45,000 persons reported in the three most densely populated municipal areas according to the Mexican Institute of Statistics and Geography (INEGI) data for 2005 (Table 1) to give a good distribution of samples. Pigeon excreta samples were collected using clean spatulas, transferred to sterile plastic bags, and properly labeled according to site and date. Samples were taken immediately to the laboratory and were processed as previously reported by Shields and Ajello with modifications described by La Pal and Baxter (Shields and Ajello, 1966; Pal and Baxter, 1985). Yeasts were isolated and characterized by phenoloxidase activity determination, capsule formation, presence of urease, sugars, nitrate assimilation, and ability to grow at 37°C (Pérez *et al.*^2003^). Species and varieties were identified using creatinine dextrose bromothymol blue thymine medium and l-canavanine glycine bromothymol blue agar. We also evaluated the color, morphology, and colonial growth in CHROMagar Candida media (Irokanulo *et al.*^1994^; Kwon-Chung *et al.*^1982^; García *et al.*^1998^). *Cryptococcus neoformans* ATCC 66031 and *Candida albicans* ATCC 14053 were used as microbiological identification controls.

**Table 1:** **Samples collected for the number of inhabitants**

Municipal zones	Population	Samples	Positive samples (C.n/C.a)
**Monterrey**	1.135.550	25	(3/3)
**Guadalupe**	678.006	15	(1/2)
**San Nicolás**	443.273	10	(1/0)
**TOTAL**	2.256.829	50	(5/5)

Total samples collected in relation to the number of inhabitants in each municipal zones.

Microbiological identification was confirmed by ITS1–ITS2 ribosomal gene sequence determination ( White *et al.*^1990^). DNA fragments were obtained by polymerase chain reaction (PCR) amplification from genomic DNA extracted using the Barth and Gaillardin method from isolated yeasts (Barth and Gaillardin, 1996). Sequences were aligned with those deposited in NCBI GenBank. Accession numbers of our nucleotide sequences are JQ794489, JQ794490, JQ794491, JQ794492, JQ794493, JQ794494, JQ794495, JQ794496, JQ794497, and JQ794498 (Table 2).

**Table 2:** **Sequences characteristics**

Isolate ID	Best alignment sequences (ID)	High similarity specie	Similarity percentage	GenBank access
**Cs1**	JN939394.1	*C. neoformans*	99%	JQ794489
**Cs2**	JN939440.1	*C . neoformans*	95%	JQ794490
Cs3	HQ231895.1	*C. albidus*	94%	JQ794491
Cs4	AB032618.1	*C. albidus*	95%	JQ794492
Cs5	HQ231895.1	*C. albidus*	99%	JQ794493
**Cs6**	JN939394.1	*C. neoformans*	98%	JQ794494
Cs7	AB032617.1	*C. albidus*	96%	JQ794495
**Cs8**	AJ560306.1	*C. neoformans*	99%	JQ794496
**Cs9**	JN939394.1	*C. neoformans*	99%	JQ794497
Cs10	AB032617.1	*C. albidus*	99%	JQ794498

Percent similarity between the ITS1–ITS2 18s ribosomal gene from genomic DNA amplification from the *Cryptococcus* spp. isolates in the metropolitan area of Monterrey, Nuevo León, Mexico and those available in GenBank by *in silico* alignment.

The phylogenetic analysis was performed using MEGA view 5.2 with the Neighbor Joining algorithm (NJ), and included 18 sequences, 8 from Genbank and 10 generated in this research.

We developed a simple regression analysis using correspondence tables to determine whether there was any link between ambient conditions of the sample and the isolation results. The data were analyzed according to the isolation results using the statistics program SPSS version 17.0.

## Results

**Figure 1:**
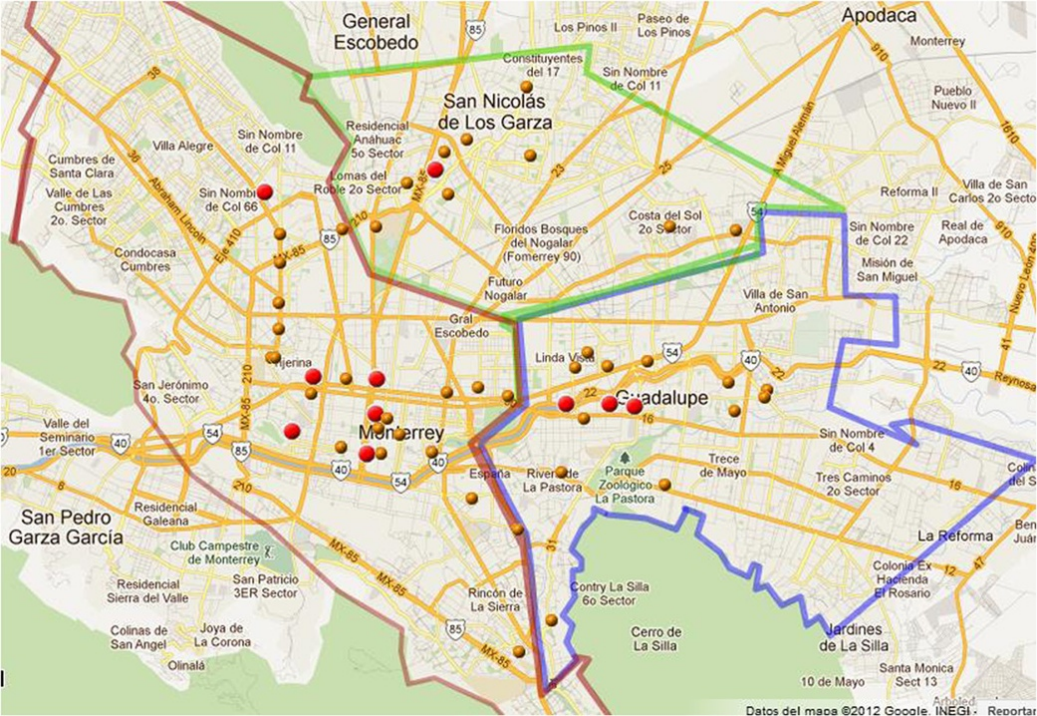
**Localization map for collected samples.** Dots indicate the location of pigeon fecal samples collected and red dots point to positive samples for *Cryptococcus* spp.

**Figure 2:**
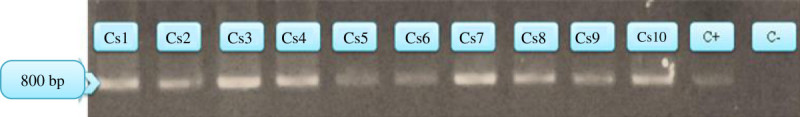
**PCR results.** PCR products obtained from the 10 *Cryptococcus* isolates. ITS fragments length were approximately 800 bp.

**Figure 3:**
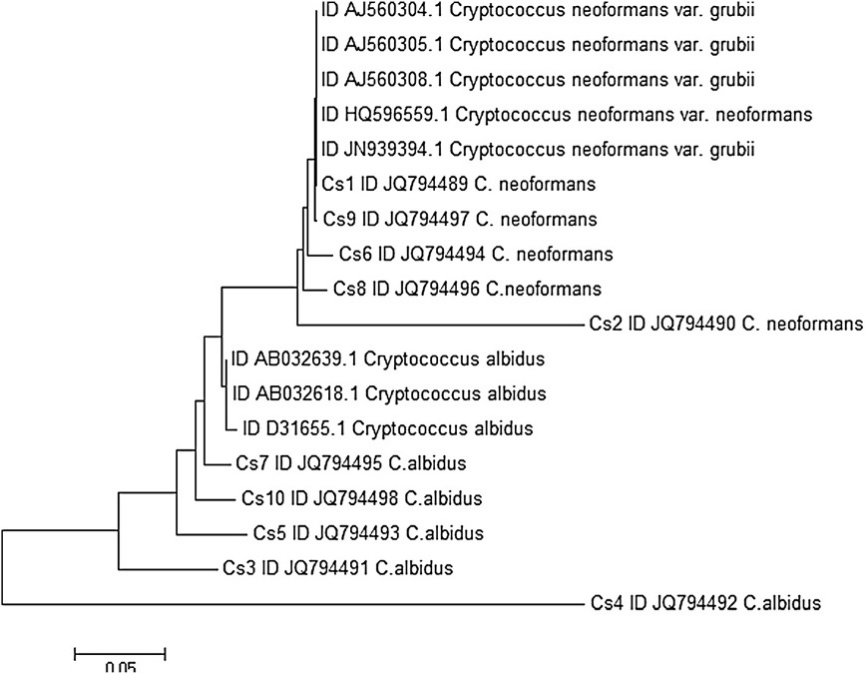
**Phylogenetic tree.** Phylogenetic relationship between 8 sequences obtained from Genbank and 10 generated in this research.

Among the 50 pigeon fecal samples obtained from three different municipal zones in the metropolitan area of Monterrey, Nuevo León, México (Figure 1), 10 *Cryptococcus* strains were isolated (20% of samples were positive) and identified using different biochemical and microbiological tests (Table 3). For each strain, sequences of approximately 800 bp were PCR amplified from the ITS1–ITS2 genomic DNA (Figure 2). Based on very high similarity between sequences from the present samples and those previously reported in GenBank (Table 2), we identified five *C. neoformans* and five *C. albidus* isolates. Most of these (three *C. neoformans* and three *C. albidus*) were found in the Monterrey city zone, which was the most populated area (Table 1). Analysis of the cladogram (Figure 3) of the 18S ribosomal sequences of our isolated strains aligned against previously reported sequences in GenBank showed that isolates 1, 9, 6, 8, and 2 are grouped with *C. neoformans* sequences and 3, 7, 5, 10, and 4 with *C. albidus*. This cladogram confirms the microbiological identification. Among 10 positive samples, 9 were isolated from shaded sites and simple regression analysis confirmed a statistical correlation (Table 2).

**Table 3:** **Isolate’s characteristics**

Isolate identification	Assays							CDBT	CGB	CMa	Microbiologic identification
	P	U	I	L	°C	N	C				
**Cs1**	+	+	+	-	+	-	+	-	-	White	*C. neoformans var. grubii*
**Cs2**	+	+	+	-	+	-	+	-	-	White	*C. neoformans var. grubii*
**Cs3**	-	+	+	+	-	+	+	-	-	Pink lavender	*C. albidus*
**Cs4**	-	+	+	+	-	+	+	-	-	Pink lavender	*C. albidus*
**Cs5**	-	+	+	-	-	+	+	-	-	Pink lavender	*C. albidus*
**Cs6**	+	+	+	-	+	-	+	-	-	White	*C. neoformans var. grubii*
**Cs7**	-	+	+	-	-	+	+	-	-	Pink lavender	*C. albidus*
**Cs8**	+	+	+	-	+	-	+	-	-	White	*C. neoformans var. grubii*
**Cs9**	+	+	+	-	+	-	+	-	-	White	*C. neoformans var. grubii*
**Cs10**	-	+	+	+	-	+	+	-	-	Pink lavender	*C. albidus*

*Cryptococcus* species identification using biochemical assays: phenoloxidase activity (P), urease activity (U), inositol (I), lactose (L), Potassium nitrate (N) and Creatinine assimilation (C), growth at 37°C (°C), Color (+) or no color changes (-) observed Creatinine dextrose bromothymol blue thymine (CDBT) agar and Canavanine-Glycine-Bromothymol Blue (CGB) agar and colonial morphotype in CHROMagar plates (CMa). *Cryptococcus* internal identification for each isolate (Cs1-Cs10).

## Discussion

Recent reports have indicated a significant prevalence of cryptococcosis in immunosuppressed patients in Monterrey city (Casillas 2012). The present study adds information about the distribution of this pathogenic yeast in the metropolitan area of Monterrey, a city with a very high population density and extreme temperature conditions in which the probability of *Cryptococcus* dissemination should be low.

Definitive *Cryptococcus spp*. identification of isolated yeasts was possible using previously reported biochemical, microbiological, and molecular characterization. From 50 pigeon excreta samples randomly collected from July to September of 2010 we identified five *C. neoformans* and five *C. albidus* isolates, representing an incidence of 20%. A similar incidence has been reported in Mexico City and in other countries such as Colombia and Perú ( Castañón Olivares et al. 2000; Ruiz et al. 1989; Rivas et al. 1999; Quintero et al. 2005; Curo et al. 2005; Colom Valiente et al. 1997;Castañol-Olivares and Lopez-Martinez, 1994). The finding that 50% of total *Cryptococcus* isolates corresponded to *C. albidus* is not surprising given the fact that 14% and 12% of pigeon excreta isolates in Seoul and Brazil correspond to this species and now it is considered an underestimated pathogen by several researchers ( Jang et al. 2011; Soares et al. 2005). Comparison of 18S RNA sequence against other reported sequences shows that the isolates form clades with their corresponding species according to the microbiological test results. However, two of them group together, mainly because of augmented intra-species sequence variability. This must be probed with a greater number of sequences, therefore it is important to continue to report *Criptococcus spp*. ITS sequences from other isolates in order to study genetic variation.

The information obtained in this research will inform the medical community about the presence of the etiologic agent of cryptococcosis in this area. Additional epidemiological studies may help set the stage for health campaigns to clean and prevent the accumulation of pigeon droppings in areas of high human traffic, such as subway stations and hospitals.

## Authors’ original submitted files for images

Below are the links to the authors’ original submitted files for images. Authors’ original file for figure 1 Authors’ original file for figure 2 Authors’ original file for figure 3
