# Supplementary figures and images for: Cryptococcus spp. isolation from excreta of pigeons (Columba livia) in and around Monterrey, Mexico
Source: Springerplus. 2013 Nov 23;2(1):632. doi: 10.1186/2193-1801-2-632 (PMC3862861; doi:10.1186/2193-1801-2-632)

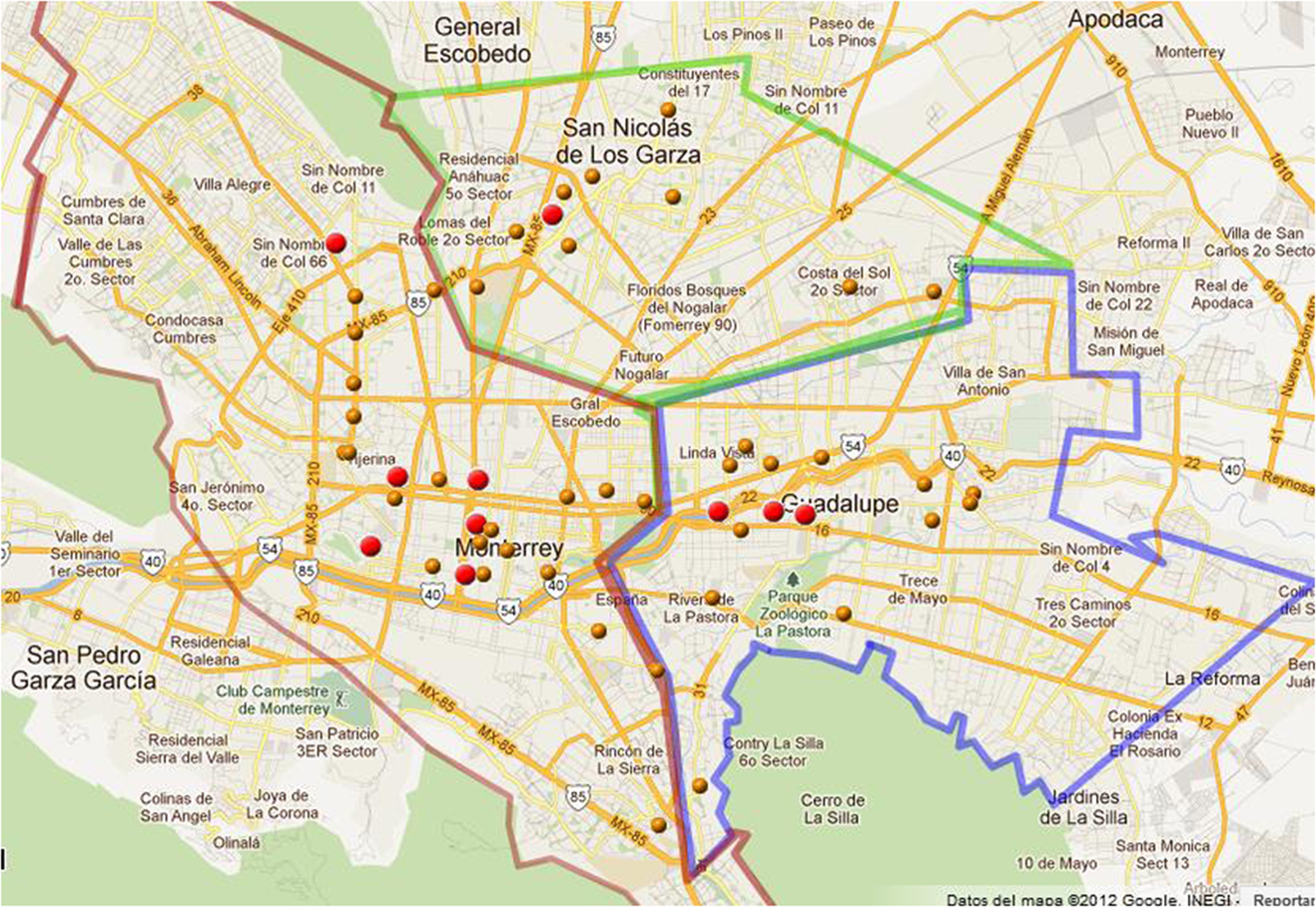

Supplement: Supplementary file 1 — Authors’ original file for figure 1 [file 40064_2013_692_MOESM1_ESM.tif]

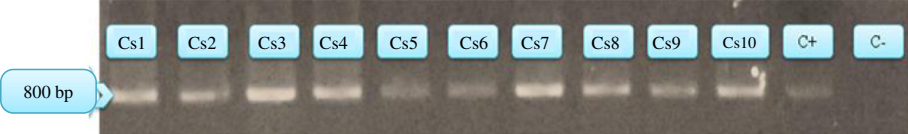

Supplement: Supplementary file 2 — Authors’ original file for figure 2 [file 40064_2013_692_MOESM2_ESM.pdf]

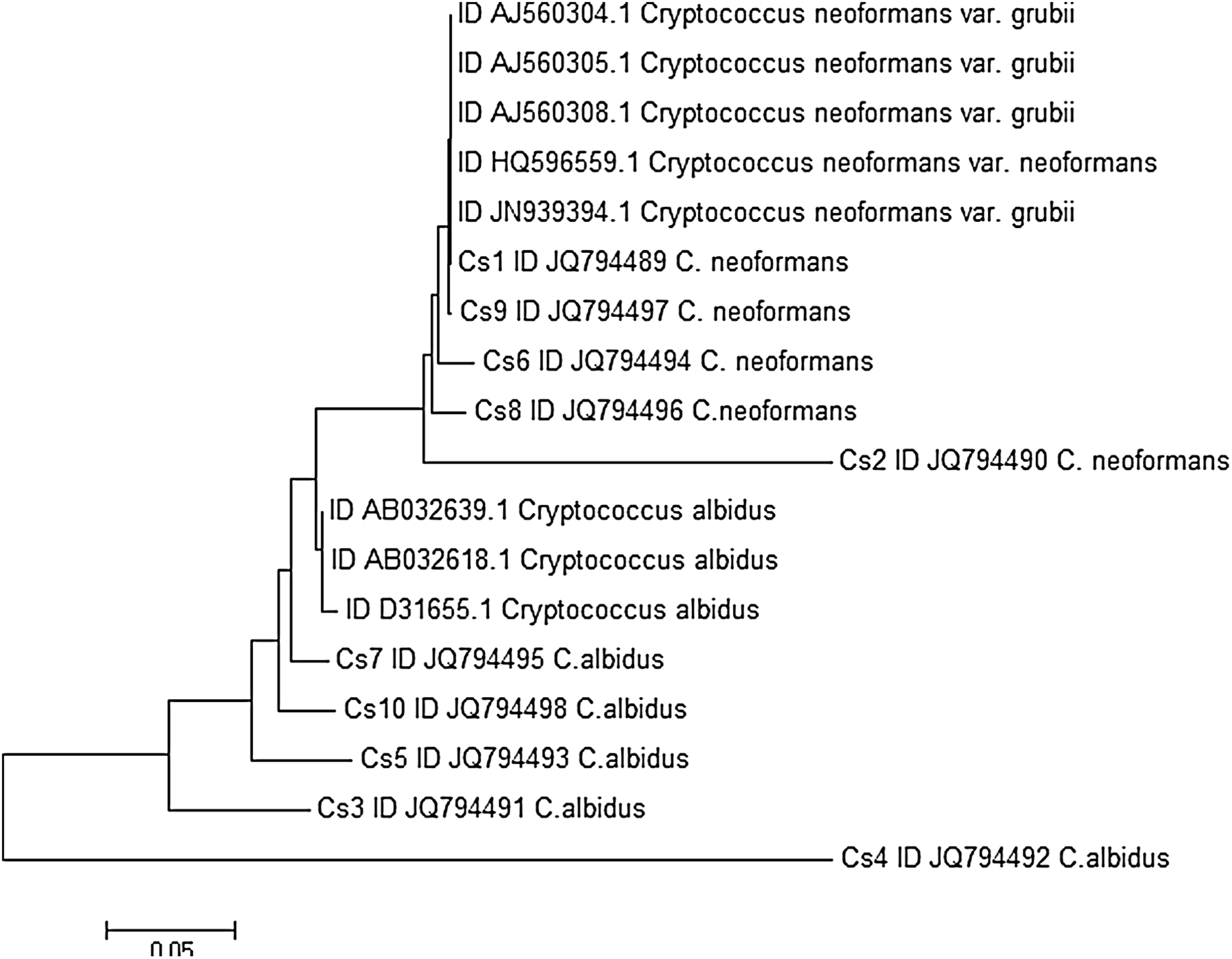

Supplement: Supplementary file 3 — Authors’ original file for figure 3 [file 40064_2013_692_MOESM3_ESM.tif]
